# *CaDHN3*, a Pepper (*Capsicum annuum* L.) Dehydrin Gene Enhances the Tolerance against Salt and Drought Stresses by Reducing ROS Accumulation

**DOI:** 10.3390/ijms22063205

**Published:** 2021-03-22

**Authors:** Yuan-Cheng Meng, Hua-Feng Zhang, Xiao-Xiao Pan, Nan Chen, Hui-Fang Hu, Saeed ul Haq, Abid Khan, Ru-Gang Chen

**Affiliations:** 1 College of Horticulture, Northwest A&F University, Yangling 712100, China; YuanchengMeng07@126.com (Y.-C.M.); 18848966687@163.com (H.-F.Z.); xiaixiao1110@163.com (X.-X.P.); nanCHEN224@163.com (N.C.); huifang1919@163.com (H.-F.H.); saeed_ulhaq@nwafu.edu.cn (S.u.H.); abidagriculturist@gmail.com (A.K.); 2 Department of Horticulture, The University of Agriculture Peshawar, Peshawar 25130, Pakistan; 3 Department of Horticulture, The University of Haripur, Haripur 22620, Pakistan; 4 Shaanxi Engineering Research Center for Vegetables, Yangling 712100, China

**Keywords:** dehydrins, *CaDHN3*, overexpression, abiotic stresses

## Abstract

Dehydrins (DHNs) play an important role in abiotic stress tolerance in a large number of plants, but very little is known about the function of DHNs in pepper plants. Here, we isolated a Y_1_SK_2_-type DHN gene “*CaDHN3*” from pepper. To authenticate the function of *CaDHN3* in salt and drought stresses, it was overexpressed in Arabidopsis and silenced in pepper through virus-induced gene silencing (VIGS). Sub-cellular localization showed that *CaDHN3* was located in the nucleus and cell membrane. It was found that *CaDHN3*-overexpressed (OE) in Arabidopsis plants showed salt and drought tolerance phenotypic characteristics, i.e., increased the initial rooting length and germination rate, enhanced chlorophyll content, lowered the relative electrolyte leakage (REL) and malondialdehyde (MDA) content than the wild-type (WT) plants. Moreover, a substantial increase in the activities of antioxidant enzymes; including the superoxide dismutase (SOD), peroxidase (POD), catalase (CAT), ascorbate peroxidase (APX), and lower hydrogen peroxide (H_2_O_2_) contents and higher O_2_^•−^ contents in the transgenic Arabidopsis plants. Silencing of *CaDHN3* in pepper decreased the salt- and drought-stress tolerance, through a higher REL and MDA content, and there was more accumulation of reactive oxygen species (ROS) in the *CaDHN3*-silenced pepper plants than the control plants. Based on the yeast two-hybrid (Y2H) screening and Bimolecular Fluorescence Complementation (BiFC) results, we found that CaDHN3 interacts with CaHIRD11 protein in the plasma membrane. Correspondingly, the expressions of four osmotic-related genes were significantly up-regulated in the *CaDHN3*-overexpressed lines. In brief, our results manifested that *CaDHN3* may play an important role in regulating the relative osmotic stress responses in plants through the ROS signaling pathway. The results of this study will provide a basis for further analyses of the function of DHN genes in pepper.

## 1. Introduction

Plants are exposed to a large number of abiotic stresses for instance, drought and salt stresses. These stresses hinder plant growth, development, yield and severe can lead to plant death [1]. To survive in the stressed environment, plants have adapted different responses, such as the plant responses at molecular and physiological levels [2]. Many stress-inducible genes with various functions have been identified in Arabidopsis, rice, and other plants, including a number of transcription factors that regulate stress-inducible gene expression. The products of stress-inducible genes function both in the initial stress response and in establishing plant stress tolerance [3].

Pepper (*Capsicum annuum* L.) is one of the important vegetable crops in the world and is appreciated due to its unique taste and rich nutritional value [4]. During the growth and development stage, the pepper plants are easier to suffer from different stresses, including salt and drought conditions. These adverse conditions may decrease pepper seedling germination, growth, and in severe cases, accelerate pepper death. DHNs can improve plants’ tolerance to abiotic stresses, prevent cell dehydration, maintain cell homeostasis, eliminate the free radicals and combine the metal ions [5,6]. Therefore, it is necessary to explore the stress resistance of plants and their adaptation to stressed conditions [7]. 

DHNs are part of group II of the late-embryogenesis-abundant (LEA) proteins. Generally, DHN proteins are regarded as a group of hydrophilic proteins which include three conserved structural domains, i.e., the K, Y, and S fragments. The K-segment contains the Lys-rich structure which shapes an α-helix in the C-terminus, that interacts with the lipid membrane or the proteins [8,9,10], while all the dehydrins have the K segments. However, some dehydrins contain the S and Y segments. According to the three conserved motifs, DHNs have been classified into five groups; namely YnSKn, YnKn, SKn, Kn, and KnS [11,12].

DHNs play an important role in tolerance to abiotic stresses, which can maintain cell stabilization, and protect macromolecules when subjected to drought, cold, salt and high-temperature stresses. Multiple DHN genes have been found in tomato, rice, banana, wheat, maize, and Arabidopsis, which are reported to enhance the cold and drought stresses [13,14,15,16,17,18]. DHNs play a valuable role in drought stress tolerance in a large number of plant species [8], such as *HbDHN1* and *HbDHN2* enhanced the plant tolerance to drought and osmotic stresses in banana [19]. Likewise, in *Sorghum bicolor*, the Y_S_K_2_ Type dehydrin (*SbDHN1*) improved protection at high temperature and osmotic stress conditions [20], the maize KS-type dehydrin (*ZmDHN13*) enhanced the tolerance of transgenic tobacco to oxidative stress. However, little is known about the function of DHNs in pepper.

Thus, exploring the role of DHN genes under abiotic stresses will contribute to the regulation mechanism of DHNs. Our previous studies found 7 DHN genes in pepper [21], and previous studies divulged that DHNs are localized in cytoplasm [22], nucleus [23], mitochondria [24] and chloroplasts [25]. Although there are many reports on DHNs, however, its regulation mechanism is still unknown. In this research, we separated a pepper Y_1_SK_2_-type dehydrin gene *CaDHN3* (CA02g06010 from pepper CM334 database), based on our previous study that it was strongly induced by salt and drought stresses [21]. Thus, the purpose of this study was to learn the functional role of the *CaDHN3* and its regulation under salt and drought stresses. The function of the *CaDHN3* was analyzed through overexpression in Arabidopsis and silencing through virus-induced gene silencing (VIGS) in pepper. The *CaDHN3*-transgenic Arabidopsis and *CaDHN3*-silenced pepper plants were subjected to salt and drought conditions. The results of this study manifested that this gene positively regulated the plants against salt and drought stresses. This study will provide useful information about the function of DHNs in pepper and other important vegetable crops.

## 2. Results

### 2.1. CaDHN3 Sub-Cellular Localization

**Figure 1:**
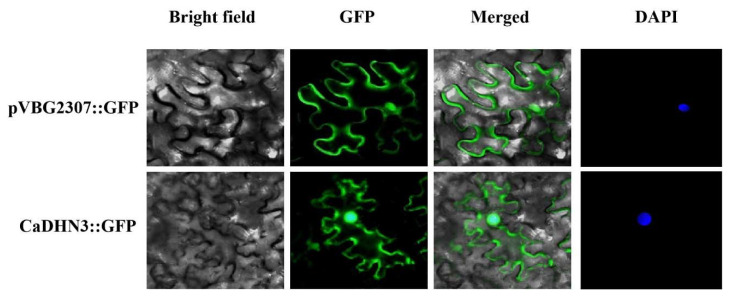
The sub-cellular localization of pepper CaDHN3. Agrobacterium tumefaciens strain GV3101 with pVBG2307:CaDHN3:GFP and pVBG2307:GFP (as control) vectors were transiently expressed in N. *benthamiana* leaves. Bars in this picture are 50 µm.

To know the sub-cellular localization of CaDHN3 protein, the fusion protein pVBG2307-CaDHN3-GFP was constructed. The GV3101 strain with pVBG2307:*CaDHN3*:GFP and pVBG2307:GFP (as control) vectors were rapidly expressed in the leaves of N. *benthamiana* plants. According to the instantaneous conversion assay, the GFP-CaDHN3 fusion protein was showed in the nucleus and the cell membrane of tobacco cells Figure 1.

### 2.2. Silencing of CaDHN3 Reduces Salt Stress Tolerance in Pepper

**Figure 2:**
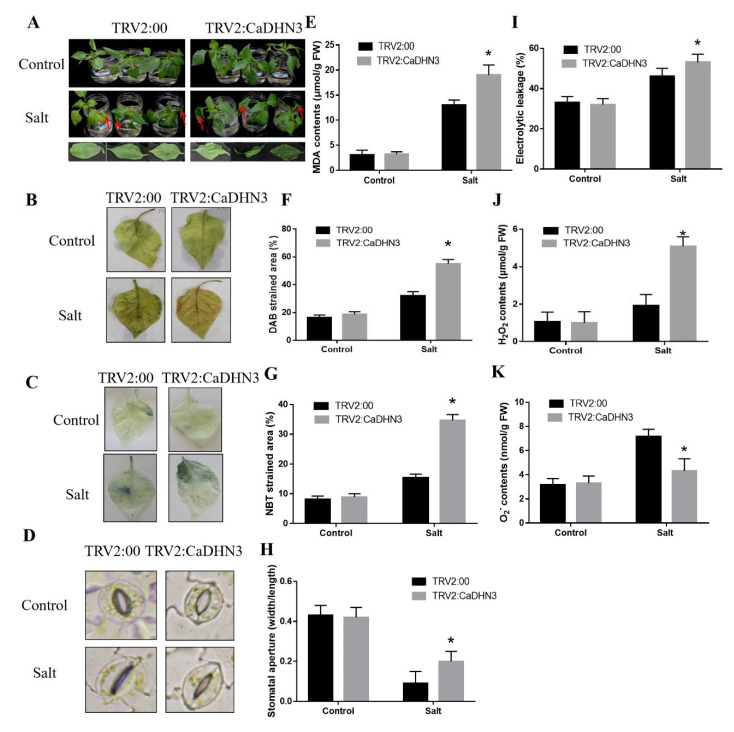
Physiological indies measurement under salt stress. (**A**) Phenotypes of silenced pepper plants under salt stress. (**B**) DAB staining. (**C**) NBT staining. (**D**) Stomatal conductance. (**E**) malondialdehyde (MDA) content. (**F**) % DAB strain areas. (**G**) % NBT strain areas. (**H**) Stomatal aperture. (**I**) Relative electrolyte leakage. (**J**) H_2_O_2_ contents (**K**) O_2_^•−^ contents. Mean and S.D. values were obtained from three independent experiments. Asterisks indicate statistical significance (* *p* < 0.05, Student’s *t*-test) compared to control.

To assess the response of *CaDHN3* in salt-stress of pepper, the VIGS assay of the *CaDHN3* was carried out in the pepper cold-resistant cultivar “P70”. After 30 days of post-inoculation, the injected pTRV2-*PDS* plants were photo-bleached Figure S1A, and the relative expression of the pTRV2:00 and pTRV2:*CaDHN3* were analyzed by qRT-PCR. The results indicated that the expression of *CaDHN3* in the silenced plants decreased 86.32%, which showed that *CaDHN3* was successfully silenced in the pepper (Figure S1B). Under control conditions, no obvious differences were noticed. However, after 3 days of salt stress (300 mM NaCl), the pTRV2:*CaDHN3* plant showed more wilting than the pTRV2:00 plants Figure 2A, Furthermore, to analyze the production of ROS in the *CaDHN3*-silenced and control (pTRV2:00) pepper plants after salt stress, the DAB and NBT staining was performed. The DAB- and NBT-stained area was substantially increased in both the pTRV2:00 and pTRV2:*CaDHN3* pepper plants Figure 2B,C but the strained areas of the pTRV2:*CaDHN3* plants were higher (~26.88–50.14%) than the pTRV2:00 Figure 2F,G. In addition, we measured the stomatal conductance of the pTRV2:*CaDHN3* and pTRV2:00 plants under salt stress Figure 2D. Under normal growing conditions, there were no noticeable changes in the stomatal aperture. However, under salt stress, the pTRV2:*CaDHN3* plants had an increased (~42%) average stomatal aperture than the control pepper plants Figure 2H. Moreover, the H_2_O_2_ contents in the pTRV2:00 plants were evidently lowered (~22.05%) than the *CaDHN3*-silenced pepper plants Figure 2J; whereas, the O_2_^•−^ content in the pTRV2:00 was significantly higher (~24.33%) than the *CaDHN3*-silenced plants (Figure 2K). These results indicated that the accumulation of ROS in the *CaDHN3*-silenced plants was higher than the pTRV2:00. Before salt stress, there was no evident difference in the malondialdehyde (MDA) contents and REL of the pTRV2:00 and *CaDHN3*-silenced plants; however, 3 days post salt stress, the *CaDHN3*-silenced plants showed higher MDA contents than the pTRV2:00 plants Figure 2E, and the *CaDHN3*-silenced plants showed higher REL contents than the pTRV2:00 plants Figure 2I. These results showed that silencing of *CaDHN3* in the pepper decreased tolerance to salt stress.

### 2.3. Knockdown of CaDHN3 Reduced Drought-Stress Tolerance in Pepper

**Figure 3:**
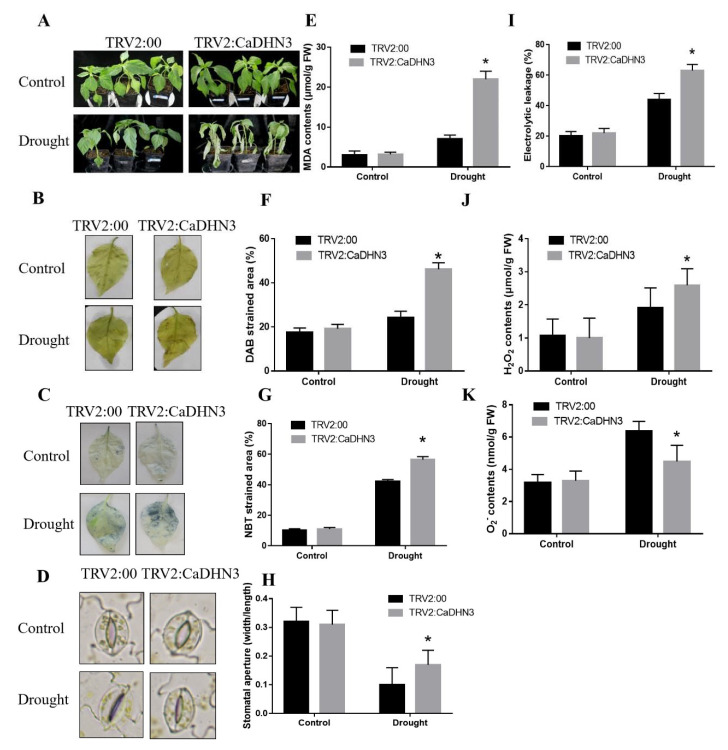
Physiological indices measurement under drought stress. (**A**) Phenotypes of silenced pepper plants under drought Scheme. (**B**) DAB staining. (**C**) NBT staining. (**D**) Stomatal conductance. (**E**) MDA content. (**F**) % DAB strain areas. (**G**) % NBT strain areas. (**H**) Stomatal aperture. (**I**) Relative electrolyte leakage. (**J**) H_2_O_2_ contents (**K**) O_2_^•−^ contents. Mean and S.D. values were obtained from three independent experiments. Asterisks indicate statistical significance (* *p* < 0.05, Student’s test) compared to control.

To study the effect of *CaDHN3* in drought-stress tolerance, the *CaDHN3* was silenced through VIGS in pepper cultivar P70. Under control conditions no obvious differences were observed in the phenotypes of control and silenced pepper plants; however, 7 days post-drought stress, the pTRV2:*CaDHN3* pepper plants were more wilted than the pTRV2:00 Figure 3A. Moreover, post-drought stress and the accumulation of ROS through NBT and DAB staining were examined in the control and *CaDHN3*-silenced pepper plants. The results showed that the ROS accumulation was higher in the pTRV2:*CaDHN3* pepper plants than the control Figure 3B,C. The DAB and NBT strained areas of the pTRV2:*CaDHN3* pepper plants were higher (~34.99–56.20%) than the control plants Figure 3F,G. Additionally, we also measured the stomatal conductance of the *CaDHN3*-silenced and control pepper plants under drought stress Figure 3D. Under normal growing conditions, there were no distinct changes in the stomatal aperture. However, under drought stress, an increased average stomatal aperture (~52.36%) was observed in the *CaDHN3*-silenced pepper plants than the control Figure 3H. The H_2_O_2_ contents in the *CaDHN3*-silenced pepper plants were significantly higher than in the control plants Figure 3J, while the O_2_^•−^ contents of the *CaDHN3*-silenced plants were markedly lower than the control plants Figure 3K. These results indicated that the accumulation of ROS in the *CaDHN3*-silenced pepper plants was higher as compared to the control plants. Similarly, before drought stress, no significant differences in the MDA contents and REL of the control and *CaDHN3*-silenced pepper plants were observed. However, after drought stress, the CaDHN3-silenced pepper plants showed higher MDA and lowered REL contents than the pTRV2:00 plants Figure 3E,I). This showed that silencing of *CaDHN3* decreased the drought stress tolerance of the pepper. 

**Figure 4:**
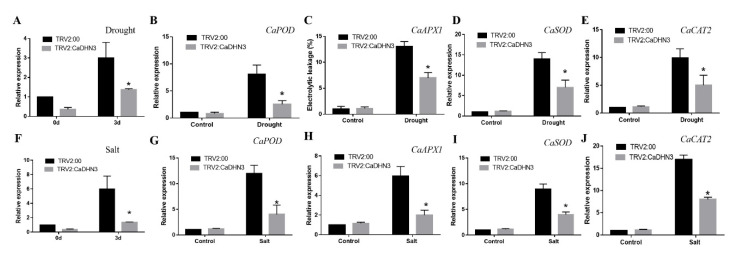
Reactive oxygen species (ROS) scavenging enzymes related gene expression after 3 days of salt stress and 7 days of drought stress. (**A**,**F**) relative expression of *CaDHN3* under drought and salt stress. (**B**,**G**) relative expression of *CaPOD* under drought and salt stress. (**C**,**H**) relative expression of *CaAPX1* under drought and salt stress. (**D**,**I**) relative expression of *CaSOD* under drought and salt stress. (**E**,**J**) relative expression of *CaCAT2* under drought and salt stress. Mean and S.D. values were obtained from three independent experiments. Asterisks indicate statistical significance (* *p* < 0.05, Student’s test) compared to control.

Furthermore, the expression of *CaDHN3* and ROS related genes *CaAPX1*(GenBank accession number: DQ002888.1), *CaCAT2* (GenBank accession number: AY128694.1), *CaSOD* (NM_001324998.1) and *CaPOD* (NM_001324997.1) were strongly induced after drought and salt treatment, the expression of *CaDHN3* in the detached leaves of control plants significantly higher than that in the detached leaves of *CaDHN3*-silenced pepper plants Figure 4A,F. After 3 days of salt stress and 7 days of drought stress, the expression of *CaAPX1*, *CaCAT2*, *CaSOD* and *CaPOD* were lower in the *CaDHN3*-silenced pepper plants than the control plants Figure 4B–E,G–J. 

### 2.4. Overexpression of CaDHN3 in Arabidopsis Increased Salt Stress Tolerance

**Figure 5:**
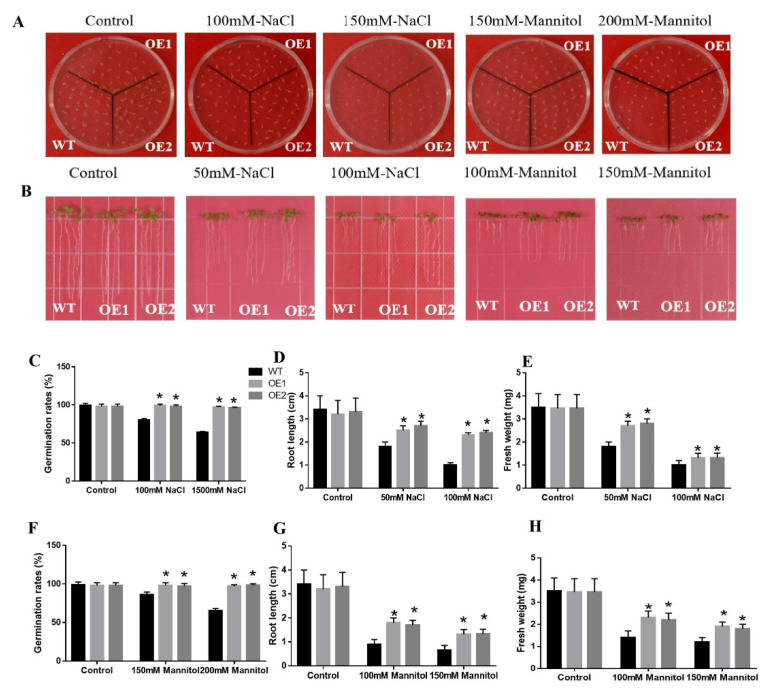
Analyses of salt and osmotic resistance of *CaDHN3*-overexpressed transgenic Arabidopsis and WT Arabidopsis plants. (**A**) Phenotypes of seed germination rates. (**B**) Root lengths. (**C**,**F**) Germination rates. (**E**,**H**) Fresh weights. (**D**,**G**) Root lengths. Mean and S.D. values were obtained from three independent experiments. Asterisks indicate statistical significance (* *p* < 0.05, Student’s test) compared to control.

To find out the role of *CaDHN3* in Arabidopsis under salt stress, the *CaDHN3*-transgenic Arabidopsis lines and WT plants were exposed to salt stress at various stages of growth. We first inspected the seed germination rates of transgenic Arabidopsis and WT plants on ½ MS medium including different concentrations of NaCl (0, 100, and 150 mM). Similarly, the root growth of the transgenic Arabidopsis and WT plants on ½ MS medium containing different concentrations of NaCl (0, and 50 mM and 100 mM) was also observed. Under normal conditions, the germination rates and root length of the transgenic Arabidopsis and the WT plants showed no significant differences, but the transgenic Arabidopsis plants showed evidently higher germination rates and root lengths than the WT plants under NaCl treatments Figure 5C,D. To further explore the role of *CaDHN3*-overexpressed Arabidopsis plants’ response to salt stress, we measured the fresh weight of the transgenic and WT seedlings. It was noticed that with the increase of NaCl concentrations, the fresh weight of the transgenic seedlings and WT gradually decreased. However, under 50 and 100 mM NaCl concentrations, the fresh weight of the transgenic seedlings was significantly higher (~40.2–42.46% and ~20.26–21.37%) than the WT seedlings Figure 5E. These results demonstrated that ectopic expression of *CaDHN3* enhanced the salt stress tolerance in Arabidopsis.

### 2.5. Overexpression of CaDHN3 in Arabidopsis Increased Drought Tolerance

To authenticate the mechanism of *CaDHN3*-overexpressing Arabidopsis plants to drought stress, the transgenic and WT plants were subjected to drought conditions at different germination and mature stages. The germination rates of the transgenic Arabidopsis and WT plants were measured on 1/2 MS solid medium with different concentrations of mannitol (0, 150, and 200 mM). The transgenic Arabidopsis plants showed markedly higher germination rates and root lengths than the WT plants under mannitol treatments Figure 5A,B. While under normal conditions, the germination rates and root length of the transgenic Arabidopsis and WT plants showed no observable differences, but under mannitol treatments, the transgenic plants revealed higher germination rates and root lengths than the WT plants Figure 5F,G. To know the role of the *CaDHN3*-overexpressed Arabidopsis plants in response to drought stress, the fresh weight of transgenic Arabidopsis seedlings and WT plants were measured. It was found that with the increase in mannitol concentrations, the fresh weights of the transgenic and WT plants were decreased, but the transgenic plants revealed higher fresh weights than WT plants Figure 5H. These results indicated that overexpression of *CaDHN3* enhanced the drought stress tolerance in Arabidopsis.

**Figure 6:**
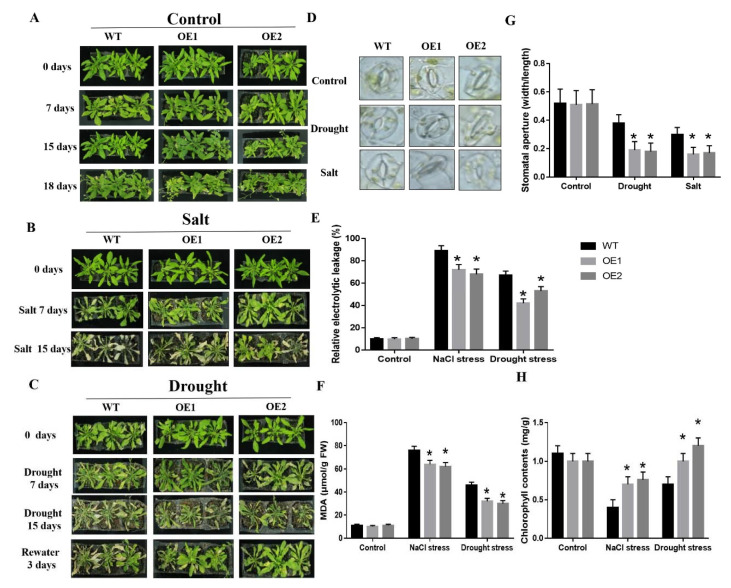
Overexpression of *CaDHN3* in Arabidopsis enhanced the resistance of Arabidopsis to salt and drought stresses. (**A**) Phenotypes under normal conditions. (**B**) Phenotypes under salt stress. (**C**) Phenotypes of under drought stress. (**D**) and (**G**) Stomatal aperture of *CaDHN3* transgenic and WT plants in response to salt and drought stress. (**E**) Electrolytic leakage rate. (**F**) MDA content. (**H**) Chlorophyll content. Mean and S.D. values were obtained from three independent experiments. Asterisks indicate statistical significance (* *p* < 0.05, Student’s test) compared to control.

Then we also determined the salt and drought stress tolerance of the transgenic and WT lines under soil-grown conditions. We found that the growth of transgenic and WT plants has no difference under normal growing conditions Figure 6A. However, after 300 mM NaCl treatment for 7 days, the leaves of the transgenic plants were gradually yellowed, but the leaves of the WT plants became shrank and even death occurred. After 300 mM NaCl treatment for 15 days, the WT plants died, but the transgenic plants survived and were pale green Figure 6B. After drought stress for 15 days, the WT plants showed serious wilting as compared to the transgenic plants. While, after re-watering for 3 days, the transgenic lines showed a higher survival and recovery rate than the WT plants Figure 6C. We also found that the chlorophyll content of the transgenic plants was significantly higher under salt (~40.46–42.39%) and drought (~20.26–24.46%) stresses than the WT plants Figure 6H. 

To further confirm the roles of *CaDHN3* in drought and salt stress tolerance, MDA contents and REL were determined under normal and osmotic stress conditions. The MDA contents and REL of the WT and transgenic plants showed no difference under normal growing conditions, but under salt stress conditions the transgenic Arabidopsis plants showed lower MDA contents and REL than the WT plants. Similarly, under drought stress the transgenic Arabidopsis plants showed lower MDA contents and REL than the WT plants Figure 6E,F. It showed that ectopic expression of that *CaDHN3* increased the salt and drought stresses tolerance in Arabidopsis.

Besides, we also measured the stomatal conductance of the transgenic and WT Arabidopsis plants under salt and drought stresses Figure 6D. Under normal conditions, the WT and transgenic plants showed no distinct difference in the stomatal aperture. However, under salt and drought stress conditions, the *CaDHN3*-overexpressed plants had a reduced (~40.0–60.44%) average stomatal aperture than the WT plants Figure 6G. These results showed that *CaDHN3* promoted drought- and salt-induced stomatal closure. 

### 2.6. CaDHN3 Overexpression Relieves ROS Accumulation

**Figure 7:**
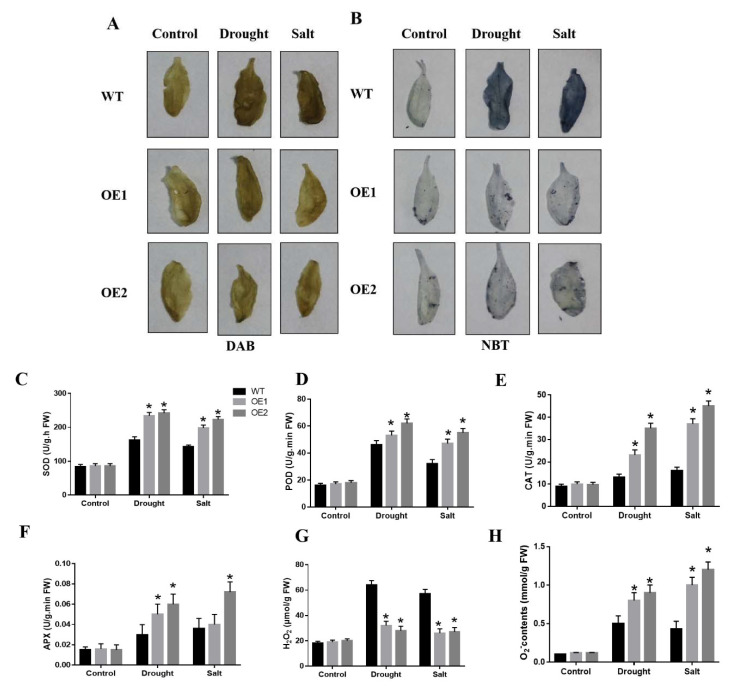
Overexpression of *CaDHN3* enhanced ROS scavenging ability in transgenic Arabidopsis. (**A**,**B**) DAB and NBT staining. (**C**) Superoxide dismutase (SOD) activity. (**D**) Peroxidase (POD) activity. (**E**) Catalase (CAT) activity. (**F**) Ascorbate peroxidase (APX) activity. (**G**) H_2_O_2_ contents. (**H**) O_2_^•−^ contents. Mean and S.D. values were obtained from three independent experiments. Asterisks indicate statistical significance (* *p* < 0.05, Student’s test) compared to control.

To know if the increased drought and salt tolerance is consisted with the ROS accumulation, we performed DAB and NBT staining also determined the H_2_O_2_ and contents, respectively. Under normal growing conditions, there were no distinct changes in the ROS accumulation of the transgenic and WT plants. However, under salt and drought stresses, the transgenic plants gathered little H_2_O_2_ and higher O_2_^•−^ contents than the WT, as little blue or brown colors of DAB or NBT staining were detected in the leaves Figure 7A,B. 

It is known that superoxide dismutase (SOD), peroxidase (POD), catalase (CAT), and ascorbate peroxidase (APX) play a vital role in ROS homeostasis during NaCl and drought stresses by scavenging ROS. Next, we measured SOD, POD, CAT and APX enzymatic activity under drought or salt stresses, the SOD, POD, CAT and APX showed significantly higher activity in the transgenic lines compared to WT plants under salt and drought treatments Figure 7C–F. Then we determined in situ accumulation of two major ROS, H_2_O_2_ and O_2_^•−^ Quantitative results also showed that cellular H_2_O_2_ contents in the overexpressing lines were lower than in the WT, however, O_2_^•−^ contents was higher in the overexpressing lines than WT Figure 7G,H. These results showed that *CaDHN3* overexpression can relieve ROS accumulation of the transgenic plants through increasing the activities of these anti-oxidative enzymes.

### 2.7. CaDHN3 Overexpression Activated the Expression of Relative Stress-Responsive Genes

**Figure 8:**
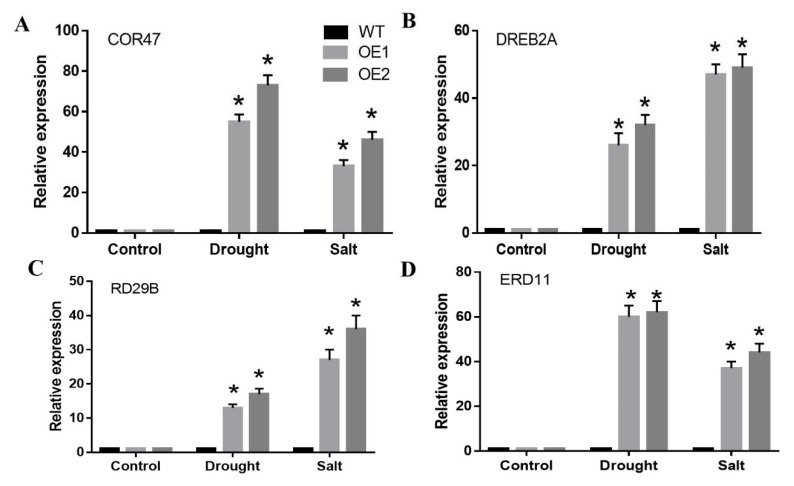
Overexpression of CaDHN3 activated the relative expression of the stress responsive genes. (**A**) Relative expression of COR47 under drought and salt stress. (**B**) Relative expression of DREB2A under drought and salt stress. (**C**) Relative expression of RD29B under drought and salt stress. (**D**) Relative expression of ERD11 under drought and salt stress. Mean and S.D. values were obtained from three independent experiments. Asterisks indicate statistical significance (* *p* < 0.05, Student’s test) compared to control.

To further understand the regulating mechanisms of *CaDHN3* in drought and salt stresses, the expression of stress-responsive genes including *COR47, DREB2A, ERD11* and *RD29B* were measured in *CaDHN3*-overexpressed and WT plants by QPCR Figure 8. In addition, the expression level of drought and salt stress-related genes *COR47*, *DREB2A*, *ERD11* and *RD29B* were strongly induced in transgenic Arabidopsis after drought and salt treatment. The expression level of the *COR47*, *DREB2A*, *ERD11* and *RD29B* genes were higher in transgenic lines than that in WT. These results suggest that *CaDHN3* positively activates drought and salt-responsive gene expression in transgenic Arabidopsis.

### 2.8. CaDHN3 Interact with CaHIRD11 Protein

**Figure 9:**
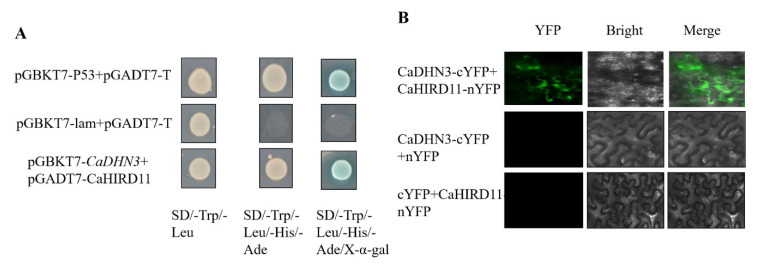
The interactions of CaDHN3 with CaHIRD11. (**A**) Interactions of CaDHN3 with CaHIRD11 by yeast two-hybrid assay, pGBKT7-53 + pGADT7-T as positive control, the pGBKT7-Lam + pGADT7-T as negative control. (**B**) BiFC assays were used to measure the interaction between CaDHN3 with CaHIRD11. Bars in this picture are 50 µm.

The ORF fragments of the proteins CaHIRD11 was inserted into pGADT7 vectors, and transferred to yeast strainsY2H Gold also containing pGBK7-CaDHN3. As shown in Figure 9, the co-transformed CaDHN3-BD and CaHIRD11-AD were grown on SD-Leu-Trp, SD/-Trp/-Leu/-His/-Ade. The results showed that CaDHN3 interacted with CaHIRD11 protein. To further confirm CaDHN3 interacts with CaHIRD11 protein, we also performed BiFC experiment in tobacco leaves. The results were consistent with Y2H, CaDHN3 interacted with CaHIRD11 protein in the plasma membrane Figure 9.

## 3. Discussion

A number of DHN genes have been found and their regulatory mechanisms have also been studied and analyzed [26,27,28,29]. The protein structure of DHNs is divided into three conserved motifs, K, Y, and S fragments. The existence or deficiency of the YSK fragments is connected with the localization of the DHN in the cell [6]. YnKn, YSK, and Kn DHNs have been found that localizes to the cytoplasm and cell nucleus [30,31]. SKn DHNs are found to localize in the plasma membrane [32,33] However, KnS DHN was found in the mitochondrial [33]. No DHN Y-segment was found to localize in the membrane, which speculates that the Y-segment does not play a valuable role in cell membrane protection [6]. *CaDHN3* contained one S fragment at the N-terminal and two K fragments at the C-terminal, thus, were named Y_1_SK_2_-type DHN. To explore the function and the regulatory pathway of different stresses, we amplified a Y_1_SK_2_ DHN gene (*CaDHN3*) from pepper, based on our previous study [21]. Many DHNs have been found that can be induced under salt and drought stresses. Overexpression of *DHNs* genes in different plant species, such as Arabidopsis, wheat and tomato enhanced the drought, salt, and cold stresses tolerance. In different stages of plant growth and development, the WT and transgenic Arabidopsis showed no differences under normal growing conditions. However, when subjected to salt and drought stresses, the transgenic Arabidopsis lines were more sensitive to both the morphological and physiological indicators than the WT. In a previous study, silencing of *CaDHNs* through VIGS, decreased cold and salt stress tolerance, and our results also showed that silencing of *CaDHN3* decreased the drought and salt stress tolerance [21], In the current study, we carried out further research of the CaDHNs for the drought and salt assay, and will offer new insights and discoveries. 

To explore the functional role of *CaDHN3*, this gene was overexpressed in Arabidopsis. Overexpression of the *CaDHN3* enhanced the drought and salt tolerance of Arabidopsis Figure 5, while silencing of *CaDHN3* in pepper decreased the drought and salt stress tolerance. The transgenic Arabidopsis lines also had higher chlorophyll contents, lower electrolyte leakage, higher germination rate and root lengths than the WT Arabidopsis plants under salt, and drought stresses Figure 4 and Figure 5. Likewise, *CaDHN3*-silenced pepper plants showed higher H_2_O_2_ contents lower O_2_^•−^ contents than the control plants. These findings demonstrated that *CaDHN3* acted as a positive regulator. 

The mechanisms of improving drought and salt tolerance in Arabidopsis might be beneficial and brings changes in the morphological, physiological and biochemical aspects. In this research, the first change was the transgenic seedlings showed longer roots than the WT Figure 4, indicating that *CaDHN3* may regulate root systems under salt and osmotic stresses, consequently improving stress tolerance. Another change could be the physiological and biochemical characteristics. When plants are subjected to adverse conditions the chlorophyll contents tend to decrease [34]. While in this study, the chlorophyll content of *CaDHN3* transgenic plants was obviously higher than the WT after salt or drought stress treatments Figure 5H. MDA content and REL are the important physiological indicators of plant resistance [35]. Therefore, we determined MDA content and REL to explore the role of *CaDHN3* overexpression in reducing the membrane damage under drought and salt conditions. The REL of WT plants was higher than the transgenic plants under salt and drought stresses Figure 5E, and the MDA content in WT plants was obviously higher than the transgenic plants under salt and drought stresses Figure 5F. In general, these results indicated that *CaDHN3*-overexpressed plants may decrease the lipid peroxidation as compared to the WT plants under salt or drought conditions. Thus, we speculate that the ectopic expression of *CaDHN3* in Arabidopsis enhanced the tolerance to salt and drought stresses through the increase of root length and maintained cell membrane stabilization.

ROS scavenging plays a vital in maintaining ROS with a low level in stressful conditions and prevents oxidative harm. Antioxidant enzymes take part in ROS detoxification and thus play a crucial part in confronting abiotic stresses. In this research, DAB, NBT staining and quantitative results showed that overexpression of *CaDHN3* caused the reduction of ROS, whereas silencing of *CaDHN3* led to the accumulation of more ROS under salt and drought stresses Figure 2, Figure 3 and Figure 7. According to the observed results, increasing antioxidant capacity and reducing ROS accumulation may be important mechanisms that improved salt and drought tolerance of *CaDHN3*-overexpressing transgenic plants. 

Plants have the enzymatic antioxidant prevent systems to protect cells from oxidative damage by scavenging ROS, such as POD, SOD, CAT and APX. *CaDHN3*-overexpressing plants had higher activities of POD, SOD, CAT and APX after salt and drought stress, suggesting that they have more enzymatic antioxidant prevent systems compared to WT plants Figure 7. These results suggest that the overexpression of *CaDHN3* impeded ROS damage under salt and drought stress by decreasing MDA and REL levels and enhancing POD, SOD, CAT and APX activities. In this study, the expression level of the *COR47*, *DREB2A*, *ERD11* and *RD29B* genes were strongly induced in the *CaDHN3* transgenic Arabidopsis than in WT under salt and drought stress Figure 8. Therefore, these results suggested that *CaDHN3* may be involved in plant salt and drought stress tolerance by modulating the expression level of stress-related genes. Along with these stress-related genes, we also measured the expression of *CaCAT2, CaSOD, CaAPX1* and *CaPOD*, which were related to ROS-scavenging enzymes after salt and drought stresses. In addition, these genes in the *CaDHN3*-silenced plants were lower than pTRV2:00 plants under salt and drought stresses Figure 3. The results of this research revealed that *CaDHN3* gene maintained the cell membrane stability, averted lipid peroxidation and reduced the accumulation of ROS, thereby playing a valuable role in salt and drought stress tolerance. Therefore, it is speculated that *CaDHN3* regulates the plant tolerance to salt and drought stresses possibly through ROS scavenging, and this needs further experimental evidence.

As reported by Xie et al. [36] that DHN MtCAS31 interacted with the ICE1 transcription factor and enhanced drought tolerance, which showed that DHNs acted as chaperons under abiotic stresses to protect plant better growth. Chaperons can impede useless proteins aggregation and form complexes with target proteins. It is difficult for DHNs to form an interaction with other proteins, explaining why some DHNs functions are based on the protein–protein interactions. 

Y2H assays indicated the interaction between CaDHN3 and CaHIRD11 protein Figure 9A. Furthermore, BiFC showed that their interaction was located on the plasma membrane Figure 9B. These results suggested that CaDHN3 enhances the tolerance against salt and drought stresses by interacting with CaHIRD11. HIRD belongs to the SK-type DHN, DHN has been proposed to play fundamental roles in the protection of cellular components. Although various stresses cause the gene expression of DHNs, the cold is a major cue for DHN accumulation in plants [6], and transgenic plants expressing DHN genes were more cold tolerant than the corresponding wild-type plants suggesting that DHNs might prevent cellular damage due to cold [37]. In Arabidopsis we found AtHIRD11 showed cryoprotective activities for lactate dehydrogenase in a hydrophobic residue-dependent manner [38], DHNs are intrinsically disordered proteins with low contents of hydrophobic amino acids, the hydrophobic residues can play important roles in the cryoprotective activities of DHNs.

At present, we know a little about the signaling pathways of pepper dehydrins. In the previous study, we found *CaDHN3* can be induced by both ABA and SA treatments [21]. So, we suggested overexpression *CaDHN3* can enhance salt and drought stresses tolerance through abscisic acid (ABA) and salicylic acid (SA) signaling pathways. In the future study, we will verify it by measuring the germination rate under ABA and SA treatments. Taken together, these findings indicated that *CaDHN3* may act as a positive regulator against salt and drought stresses by reducing ROS accumulation. This research will provide a base for further study to understand the role of the *DHN* genes in solanaceous and other crops for adaptability to different stresses.

## 4. Materials and Methods

### 4.1. Plant Materials and Growth Conditions

The Arabidopsis wild type (WT) Col-0 and pepper cold resistance cultivar “P70” were provided by the College of Horticulture, Northwest A&F University, Yangling China. The transgenic Arabidopsis and pepper plants were grown in a growth chamber at 22 °C (day for 16 h)/18 °C (night for 8 h) and a relative humidity of 70%. The T3 transgenic Arabidopsis and WT seeds were sterilized in 75% (*v*/*v*) ethanol for 30 s, treated with 5% (*v*/*v*) NaClO for 10 min, then four washes with water. The seeds were vernalized at 4 °C for 1 d, then placed on 1/2 MS medium solidified.

### 4.2. RNA Extraction and QPCR Analysis

Plant total RNA was extracted according to manufacturer’s protocol (Tian Gen, Xi’an, China), while the qRT-PCR and single cDNA synthesis were proceeded as described by Guo et al. [39]. The iQ5.0 Bio-Rad iCycler thermocycler (BioRad, Hercules, CA, USA) was used for QPCR. The pepper ubiquitin-binding gene *CaUBI3* was used as reference in pepper and Arabidopsis *Atactin2* was used as reference in Arabidopsis. The relative expression levels were measured through the 2^−ΔΔCT^ method [40], and the experiments were conducted with three biological replicates. All the primers used for the qRT-PCR were shown in the supplementary Table S1.

### 4.3. VIGS of Pepper CaDHN3

The VIGS assay was conducted according to our previous study [41]. Briefly, a 300-bp fragment of the 3′-untranslated region of CaDHN3 was inserted into the pTRV2 vector for the construction of recombinant plasmid pTRV2: *CaDHN3*. Subsequently, through freeze–thaw method the pTRV1, pTRV2 (negative control), pTRV2: *CaPDS* (positive control) and pTRV2: *CaDHN3* was transformed into an Agrobacterium tumefaciens strain (GV3101). All the above-mentioned vectors (pTRV2, pTRV2-*CaPDS* and pTRV2-*CaDHN3*) were mixed at a 1:1 ratio with A. tumefaciens carrying pTRV1. Pepper plants cotyledons were infiltrated with inocula of agrobacterium suspensions (OD600 = 1.0) through 1.0 mL sterilized needleless syringe. The injected plants were grown at 18 °C in zan entirely dark environment for 2 days, and then transfer into normal growing conditions (22 °C at day for 16 h/18 °C at night for 8 h) with 50% relative humidity. About 30 days later, the control pepper plants (pTRV2:*CaPDS* and pTRV2:00) and silenced plants (TRV2:*CaDHN3*) were analyzed for calculating the silencing efficiency.

### 4.4. Transient Expression in Arabidopsis Plants

To obtain the transgenic Arabidopsis plants, the full-length of *CaDHN3* was obtained from pepper cDNA and cloned into the pVBG2307 vector and the recombinant plasmid pVBG2307-*CaDHN3* was constructed. Transgenic lines were developed through the floral dipping methods and screened by 1/2MS solid medium accompanied by 50 mg/L kanamycin, and subsequently, the T3 generations were used for further experiments.

### 4.5. Salt and Drought Assays

For analysis of salt and drought stress tolerance, two transgenic lines and the WT plants were selected. The WT and transgenic lines were grown on MS medium having NaCl (0, 100 mM, 150 mM) and mannitol (0, 150 mM, 200 mM) respectively, and assessed for seed germination. The root length and the fresh weight of the WT and transgenic lines of the above-mentioned treatments were determined 7th days, while plants growing condition was 16 h light/8 h dark cycle at 22 °C/18 °C.

For salt stress, the control (pTRV2:00) and silenced (pTRV2:*CaDHN3*) pepper plants were treated with 300 mM NaCl for 3 days. For drought analysis, plants were exposed to drought stress without water for 7 days. Then photographs were taken and the collected samples were stored at −80 °C for further use.

To further understand the response of transgenic Arabidopsis to salt and drought stress, about 3-week-old T3 transgenic and WT Arabidopsis lines were treated with salt or drought stresses. For salt treatment, plants were watered with 300 mM NaCl for 15 days at least 30 plants for each line and watering with salt solution was carried out at 3-day intervals. For drought treatment, plants suffered from drought stress by going without water for 15 days. At least 30 plants for each line were taken and then re-watered for 3 days. Phenotypic changes in Arabidopsis plants were found and photographed during salt and drought treatment periods. Samples of biochemical parameters measurement were measured 10 days after salt and drought treatments. Normal conditions grown plants were used as a positive control.

### 4.6. Measurement REL, Chlorophyll Content and MDA Content

The REL was estimated as described by Dionisio-Sese and Tobita [42]. Total chlorophyll was tested spectrophotometrically. Leaves were collected and incubated in 95% ethyl alcohol and absorbance was measured at 663 and 646 nm, as described by Lichtenthaler and Wellburn et al. [43]. The MDA contents were measured according to the method of Campos et al. [44] with minor modifications. The crude enzyme used for MDA contents was extracted by 10% trichloroacetic acid (TCA). Then, 2 mL of the crude enzyme extract was mixed with 2 mL of 0.6% thiobarbituric acid (TBA), boiled for 10 min, quickly cooled, and centrifuged at 12,000× *g* for 10 min. Absorbance was analyzed at 600, 532, and 450 nm. 

### 4.7. Measurement of H_2_O_2_ and O_2_^•−^ Contents, Histochemical Detection of ROS and Antioxidant Enzymes Activities

H_2_O_2_ content was determined according to Chakrabarty et al. [45]. Briefly, the samples were ground with 0.1% TCA (*w*/*v*). After grinding, the homogenate was centrifuged at 12,000× *g* for 15 min, the supernatant 0.5 mL was added to 2 mL 1 M KI and 0.5 mL 100 mM potassium sulfate buffer and kept in dark for 1 h. The absorption value at 390 nm was determined. O_2_^•−^ contents were estimated as described by Ke et al. [46]. The SOD and POD activity was determined as previously described by Stewart and Jariteh et al. [47,48]; the CAT activity was assayed using ultraviolet spectrophotometry [49]; the APX activity was assayed as previously described Mittova et al. [50].

### 4.8. NBT and DAB Staining

To detect the H_2_O_2_ and superoxide (O_2_^•−^) under salt and drought stresses, 3,3′-diaminobenzidine (DAB) and nitro-blue tetrazolium (NBT) staining were carried out. After staining, the leaves were soaked in 75% ethanol. After bleaching, images were taken immediately as anteriorly described by Able et al. [51]. The quantification of the DAB and NBT strained areas was obtained using the method described by Sekulska-Nalewajko et al. [52].

### 4.9. Measurement of Stomatal Aperture

Stomatal apertures after salt and drought stress were measured as described by Zhang et al. [53]. The 3-week-old T3 transgenic and WT plants were exposed to 300 mM NaCl and without water for 7d, respectively. Plants under normal conditions were regarded as a control. The leaves from the same location were chosen and then immediately images were taken. The stomatal apertures were determined by using Image J software (Rawak Software Inc., Stuttgart, Germany). Stomatal aperture values of at least 50 stomata were measured.

### 4.10. Sub-Cellular Localization, Yeast Two-Hybrid (Y2H) and Bimolecular Fluorescence Complementation (BiFC) Assay

Sub-cellular localization was analyzed as described by Zhang et al. [45]. The transcriptional activity of the CaDHN3 protein was analyzed with the yeast two-hybrid assay. For Y2H assay the cDNA fragments of CaDHN3 were cloned into pGBKT7 vectors. Similarly, cDNA fragments of CaHIRD11 were cloned into pGADT7 vectors. The Co-transformed CaDHN3-BD and CaHIRD11-AD were spotted in SD-Leu-Trp, SD/-Trp/-Leu/-His/-Ade. Yeast growth was performed at 30 °C for about 3–5 days to authenticate the interactions between CaDHN3 and CaHIRD11 proteins.

For BiFC assays, CaDHN3 was linked to the C-terminal of YFP; the dehydrin gene of CaHIRD11 was fused to the N-terminal of YFP. We performed the recombination plasmid transform into GV3101, which was then injected into tobacco leaves. The fluorescence was found after 48 h by fluorescence microscopy (Olympus Corporation, Tokyo, Japan).

### 4.11. Statistical Analysis

Data were analyzed through a *t*-test. The mean ± standard deviation (SD) originates from the average of three biological replicates and remarkable differences compare to the control group are marked at * *p* < 0.05.

## 5. Conclusions

**Figure 10:**
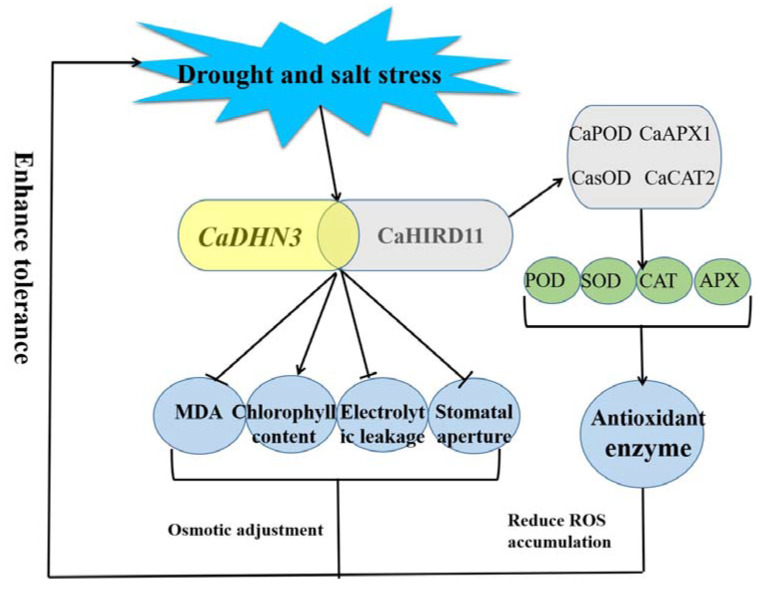
Schematic illustration of the mechanism of salt and drought tolerance regulated by *CaDHN3* in pepper.

In conclusion, we isolated a *DHN* gene (*CaDHN3*) from pepper. Ectopic expression of the *CaDHN3* in Arabidopsis enhanced resistance to salt and drought stresses through increase in the seedling root length, germination rate and protection of the cell membrane, while silencing of the *CaDHN3* decreased the salt and drought stress tolerance by reduced ROS scavenging and through lowered expression of stress- and antioxidant-related genes and antioxidant enzymes activities. According to the BiFC results, we found CaDHN3 interacted with CaHIRD11 in the plasma membrane. Further studies are suggested to focus on the regulatory mechanism of *CaDHN3* in better understanding the molecular mechanisms of abiotic stress tolerance Figure 10.

## Data Availability

Not applicable.

## Supplementary Materials

The following are available online at https://www.mdpi.com/1422-0067/22/6/3205/s1, Figure S1: The phenotypes and analysis of *CaDHN**3* expression of silencing pepper plants, Table S1: Primers were used for the qRT-PCR.
