# Supplementary material for: CaDHN3, a Pepper (Capsicum annuum L.) Dehydrin Gene Enhances the Tolerance against Salt and Drought Stresses by Reducing ROS Accumulation
Source: Int J Mol Sci. 2021 Mar 22;22(6):3205. doi: 10.3390/ijms22063205 (PMC8004091; doi:10.3390/ijms22063205)
Supplement: Supplementary file 1 [file ijms-22-03205-s001.pdf]

Supplementary materials

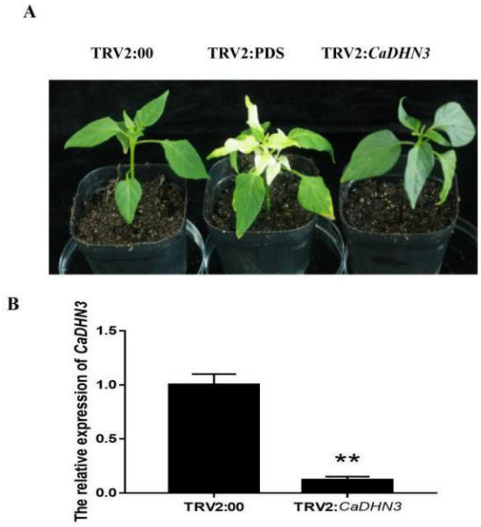

Figure S1. The phenotypes and analysis of *CaDHN3* expression of silencing pepper plants**. A**: The phenotypes of silencing pepper plants; **B**: The relative expression of *CaDHN3* of silencing pepper plants.Mean and S.D. values were obtained from three independent experiments. Asterisks indicate statistical significance (** $p < 0.01$, Student' s test) compared to control.

Table 1. Primers were used for the qRT-PCR.

Order Number	Primer Abbreviation	Primer Sequence (5′–3′)
1	CaAPX-F	AGAGGACAAGCCAGAACCAC
2	CaAPX-R	CCTTGTCTGATGGCAACTGT
3	CaCAT2-F	GAAGCCAAATCCTAAGTCCC
4	CaCAT2-R	CCAACTCGGATTGCCTCTT
5	CaSOD-F	TATGGAGCCTTAGAACCTGC
6	CaSOD-R	CCATTGAACTTGATAGCACCT
7	CaPOD-F	TCCTCCTCCTACTTCTAACC
8	CaPOD-R	ACAGACCTCTTTTGCTCACT
9	CaSOS1-F	GTTCGTGTCTCGTTTCCGC
10	CaSOS1-R	TCAAATCGGTCTGAACAGCATC
11	CaActin2-F	TCCACCTCTTCACTCTCTGCTC
12	CaActin2-R	TGACCCATCCCTACCATAACAC
13	RTCaDHN3-VIGS-F	GAGTACCTGTACCAGCAGGA
14	RTCaDHN3-VIGS-R	TAATCTTCTCCTTTCTCCCC
15	AtActin2-F	GGTAACATTGTGCTCAGTGGTGG
16	AtActin-R	AACGACCTTAATCTTCATGCTGC
17	AtCOR47-F	CAGTGTCGGAGAGTGTGGTG
18	AtCOR47-R	ACAGCTGGTGAATCCTCTGC
19	AtDREB2A-F	ATGGCAGTTTATGATCAGAGTGGAGATAGAAAC
20	AtDREB2A-R	TCATACAACCCTTCTTCGACCCTTTCGCAGGTA
21	AtRD29B-F	GAAGAAGACAACGGCTACAAAGGAG
22	AtRD29B-R	CCGAAAACCCCATAGTCCCAAC
23	AtERD11-F	5'ATCTCTTCCCTGAACAACCA3'
24	AtERD11-R	5'GCGAGAACAGCGACAACAT3'
